# Patient awareness of long‐term cardiovascular and metabolic disease risks after hypertensive disorders of pregnancy in Japan

**DOI:** 10.1111/jog.16183

**Published:** 2024-12-11

**Authors:** Takafumi Ushida, Sho Tano, Seiko Matsuo, Kazuya Fuma, Kenji Imai, Hiroaki Kajiyama, Tomomi Kotani

**Affiliations:** ^1^ Department of Obstetrics and Gynecology Nagoya University Graduate School of Medicine Nagoya Japan; ^2^ Division of Reproduction and Perinatology, Center for Maternal‐Neonatal Care Nagoya University Hospital Nagoya Japan

**Keywords:** cardiovascular disease, hypertensive disorders of pregnancy, metabolic disease, postpartum care, preeclampsia

## Abstract

**Aim:**

Given the increasing recognition of the importance of postpartum follow‐up care for women with a history of hypertensive disorders of pregnancy (HDP) to mitigate their future risk of cardiovascular and metabolic diseases, here we aimed to evaluate the current status of postpartum follow‐up care in Japan and explore the challenges to its implementation.

**Methods:**

A web‐based survey was conducted using a smartphone application among postpartum women between March and May 2024 to assess their knowledge of HDP‐related future risk and postpartum follow‐up care.

**Results:**

A total of 880 valid responses were obtained, 73 (8.3%) of which were from women with a history of HDP. Of them, 56.2% were aware of the heightened risk of cardiovascular disease and even fewer knew about the risks of metabolic syndrome (37.0%) and the preventive use of low‐dose aspirin (12.3%); in fact, 31.5% reported receiving no information about their risk or preventive measures from healthcare providers. Furthermore, 43.8% did not consult specialists or attend regular checkups after their 1‐month checkup. Among women with a history of HDP, those who received information and guidance were more likely to implement behavioral changes than those who did not.

**Conclusions:**

Patient awareness level of HDP‐related risk was low and the information provided by their healthcare professionals was insufficient, indicating that postpartum follow‐up care in Japan is not satisfactory. This study highlights the need for improved educational strategies and systematic follow‐up protocols to ensure that women are adequately informed and supported in managing their long‐term health risks.

## INTRODUCTION

Hypertensive disorders of pregnancy (HDP), including gestational hypertension and preeclampsia, is significant health concerns affecting 5%–10% of pregnant women worldwide.^1^ HDP, particularly preeclampsia, contributes to adverse maternal complications (e.g., placental abruption and HELLP [hemolysis, elevated liver enzymes, low platelet count] syndrome) and various end‐organ dysfunctions.^1^, ^2^ Importantly, the impact of HDP is not transient during pregnancy but can be lifelong, as recent long‐term cohort studies reported that women with a history of HDP are at an increased risk of developing cardiovascular and metabolic diseases later in life.^3^, ^4^, ^5^, ^6^, ^7^

Pregnancy causes adaptive changes in cardiac and metabolic function.^8^, ^9^ However, failure to achieve normal adaptation due to physiological cardiometabolic stress contributes to the development of HDP.^1^, ^10^ As pregnancy can reveal underlying and previously unrecognized cardiovascular and metabolic vulnerabilities, it is often referred to as a “stress test” for future cardiovascular diseases (CVDs).^11^ In fact, the postpartum period may offer a unique opportunity to educate women with HDP about potential health risks and implement strategies aimed at minimizing future health consequences.^12^, ^13^, ^14^

Despite the recognition of these risks, the establishment of comprehensive postpartum care programs to minimize long‐term risks in women with a history of HDP remains insufficient.^14^, ^15^, ^16^, ^17^ Challenges remain, including insufficient awareness regarding long‐term HDP‐related consequences among patients and healthcare providers, a lack of evidenced‐based follow‐up protocols, and insufficient human resources because postpartum care requires multidisciplinary management.^18^, ^19^ In Japan, the need for comprehensive postpartum care is gradually being recognized; however, a health system that fulfills postpartum care needs to be established. Furthermore, detailed data are lacking regarding women's knowledge about their increased future post‐HDP risks, their knowledge of the safety of antihypertensive drug use during breastfeeding and other preventive measures, and whether they receive appropriate guidance and follow‐up care to manage these risks. This gap in patient awareness hinders the efficacy of targeted interventions aiming to improve their long‐term health outcomes.^20^, ^21^, ^22^

To address this gap, this study aimed to assess the current status of postpartum follow‐up care for women with a history of HDP in Japan, identify gaps in knowledge and practice, and propose strategies to improve postpartum care. We conducted a web‐based survey targeting postpartum women to collect data about their knowledge of long‐term HDP‐related risks, the information and guidance they received from their healthcare providers, the feasibility and adoption of recommended lifestyle modifications, and their long‐term postpartum follow‐up. By comparing the responses of women with versus without a history of HDP, we aimed to identify specific problems and areas for improvement in patient education and healthcare practices.

This study may provide a clearer understanding of current practices in Japan regarding awareness levels and postpartum care for women with a history of HDP. By addressing the issues highlighted herein, our study may help establish postpartum care protocols and improve postpartum follow‐up care in Japan, ultimately enhancing long‐term health outcomes in women affected by HDP.

## METHODS

### Study design and participants

A cross‐sectional web‐based survey was conducted between March and May 2024 to assess awareness and follow‐up care practices among postpartum women in Japan with a particular focus on those with a history of HDP. Eligible participants included women who had given birth at least once in Japan. The participants were recruited using a smartphone application dedicated to maternal health. The survey included questions on demographic information, knowledge of HDP‐related risks, information and guidance received from healthcare providers, the feasibility and adoption of recommended lifestyle modifications, and long‐term postpartum follow‐up.

### Participant recruitment

Participants were recruited through the “Baby Plus” smartphone application created for pregnant and postpartum women in Japan. Recruitment messages and links to the survey on postpartum care were sent between March and May 2024 to women with versus without a history of HDP. No data were available on whether the participants were pregnant or in the postpartum period at the time of the survey. All responses were collected anonymously.

### Ethical considerations

This study was approved by the Ethics Committee of Nagoya University Hospital (approval no. 2023‐351; approval date: December 18, 2023). All participants provided informed consent electronically at the beginning of the survey after reading the informed consent form and were given the option to withdraw from the study at any time.

### Data collection

Two separate questionnaires were administered to women with versus without a history of HDP. The questionnaire administered to women with a history of HDP included the following sections: demographic information (five questions), awareness assessment (seven questions), information provision and guidance (five questions), feasibility and adoption of recommended lifestyle modifications (three questions), and long‐term follow‐up (four questions). The questionnaire administered to women without a history of HDP included the following sections: demographic information (four questions), awareness assessment (seven questions), feasibility of lifestyle modifications (two questions), and long‐term follow‐up (one question). Detailed survey questions for and answers from women with versus without a history of HDP are provided in Data S1 and S2, Supporting Information, respectively.

### Outcome measures

The primary outcome measures in this study were seven key aspects of HDP‐related awareness (cardiovascular risk, metabolic risk, cognitive impairment risk, recurrence risk, safety of antihypertensive drugs use, preventive use of low‐dose aspirin, and importance of lifestyle modifications) among women with a history of HDP. The secondary outcome measures included the following: information and guidance provided by healthcare providers: assessment of the items, timing, duration, and type of healthcare provider (e.g., obstetricians, midwives, and nurses) who provided information and guidance regarding HDP‐related risks and preventive measures; feasibility and adoption of lifestyle modifications: evaluation of the participant's ability to adopt recommended lifestyle modifications; stages of behavioral change: identification of the participant's current stage in the behavioral change process using the Stages of Change model,^23^ which comprises five stages—pre‐contemplation, contemplation, preparation, action, and maintenance—that reflect participant readiness to adopt and sustain health‐related behaviors; postpartum follow‐up: analysis of follow‐up care practices, specifically the rate of specialist consultations or regular checkup attendance after the initial 1‐month postpartum visit; and interest in interpregnancy care: evaluation of the participant's desire to attend an interpregnancy care program to discuss future health risks and preventive measures. Some of these outcome measures were compared between women with versus without a history of HDP and between women who were versus were not provided information and guidance by healthcare providers.

### Data analysis

Descriptive statistics were used to summarize the participants' characteristics and responses to the survey questions. Frequencies and percentages were calculated for categorical variables. Intergroup differences in categorical variables were assessed using the chi‐squared test or Fisher's exact test. The statistical analyses were performed using SPSS version 29 software (IBM Corp., Armonk, NY, USA).

## RESULTS

**FIGURE 1:**
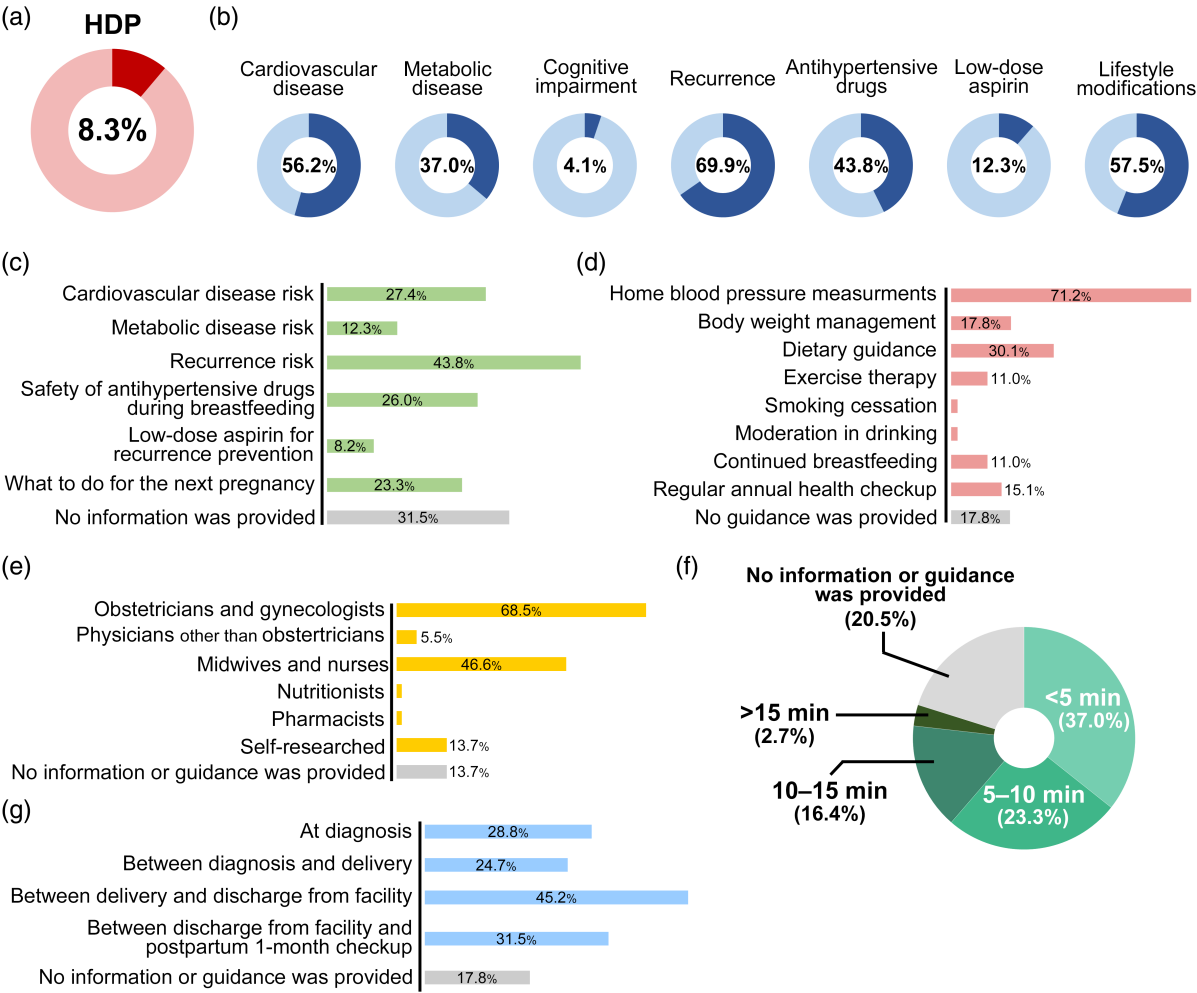
Patient awareness of hypertensive disorders of pregnancy as well as information and guidance they received from healthcare providers. (a) Ratio of women with a history of hypertensive disorders of pregnancy among the survey participants. (b) Patient awareness of hypertensive disorders of pregnancy. (c) Information about hypertensive disorders of pregnancy provided by the healthcare providers. (d) Guidance about recommended postpartum lifestyle modifications provided by healthcare providers. (e) Healthcare provider types who provided information and guidance to patients. (f) The duration of information and guidance provided by healthcare providers. (g) The timing of information and guidance provided by healthcare providers. Multiple answers were allowed for questions pertaining to (c) to (e) and (g).

A total of 880 valid responses were received, 73 (8.3%) of which were from women with a history of HDP (Figure 1a). The numbers of answers to each question are shown in Data S1 and S2.

Among women with a history of HDP, the awareness of long‐term health risks and HDP‐related knowledge was as follows (Figure 1b): CVD risk (56.2%), metabolic disease risk (37.0%), cognitive impairment risk (4.1%), HDP recurrence risk (69.9%), safety of antihypertensive drugs use during breastfeeding (43.8%), preventive use of low‐dose aspirin to reduce HDP recurrence risk (12.3%), and importance of lifestyle modifications to reduce future disease risks (57.5%). Awareness of this knowledge was similar between women with versus without a history of HDP; however, the former were more likely to be aware of the safety of antihypertensive drug use during breastfeeding (43.8%) than the latter (18.7%; *p* < 0.01) (Figure 1b, Figure S1a).

Figure 1c shows the HDP‐related information provided by healthcare providers at maternity facilities. Notably, 31.5% of the participants reported not receiving information from their healthcare providers regarding risks or preventive measures. Figure 1d shows the preventive measures recommended by the healthcare providers. Most women with a history of HDP received information and guidance from obstetricians and gynecologists (68.5%) or midwives and nurses (46.6%) (Figure 1e). Although “<5 min” and “between delivery and discharge from your maternity facility” were the most frequent answers, the timing and duration of the explanation and guidance from their healthcare providers varied (Figure 1f,g).

**FIGURE 2:**
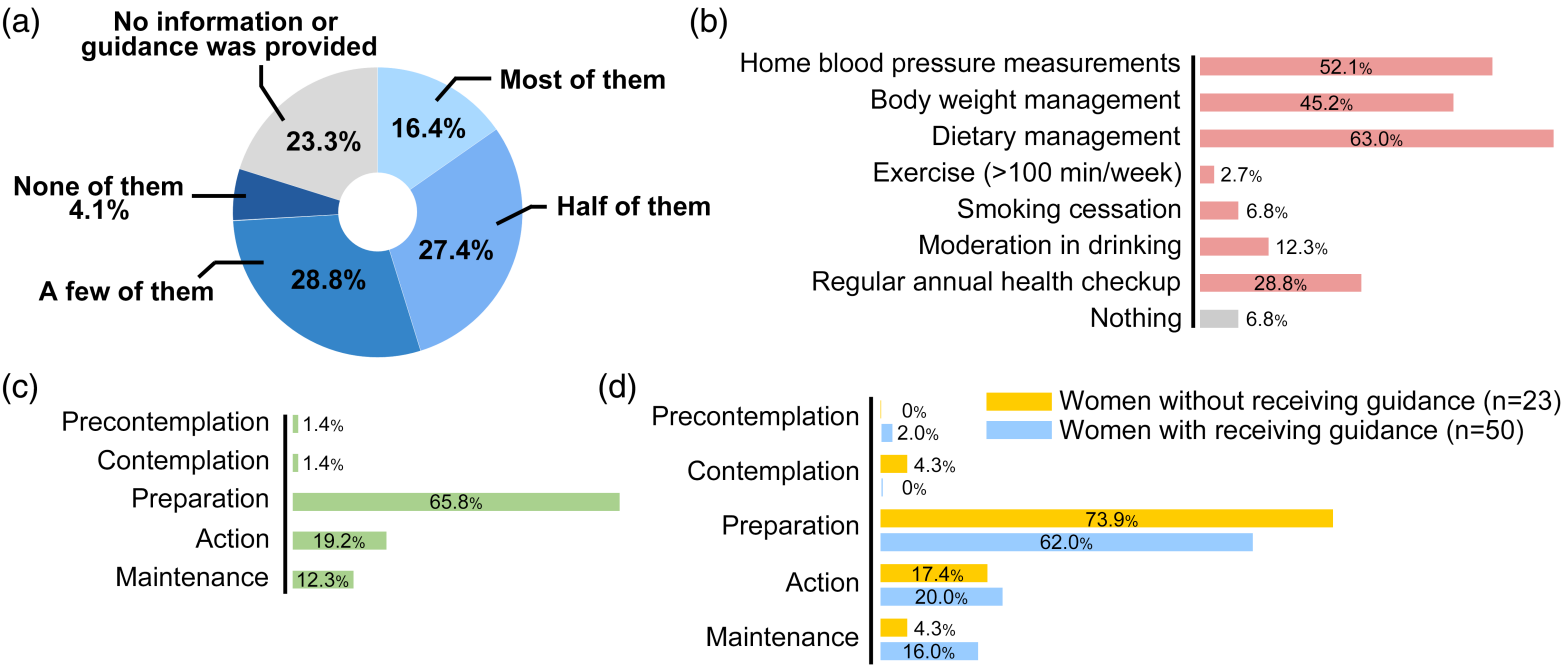
Feasibility and adoption of lifestyle modifications and stages of behavioral change among women with a history of hypertensive disorders of pregnancy. (a) Extent to which women with a history of hypertensive disorders of pregnancy were able to adhere to the recommended lifestyle modifications. (b) Lifestyle modifications currently adopted by women with a history of hypertensive disorders of pregnancy. (c) Stages of behavioral change among women with a history of hypertensive disorders of pregnancy. (d) Stages of behavioral change of women who did versus did not receive any support. Multiple answers were allowed for questions pertaining to (b).

Figure 2a shows the extent to which women with a history of HDP were able to adhere to the recommended preventive measures. Figure 2b shows the recommended lifestyle modifications that they adopted. Approximately half of the participants continued to perform home blood pressure measurements and manage their body weights and diets. Figure S1b shows the lifestyle modification items that women without a history of HDP considered feasible during child‐rearing. Women with a history of HDP were more likely to be in the active (19.2% vs. 8.9%) and maintenance (12.3% vs. 8.0%) stages than those without a history of HDP (Figure 2c, Figure S1c). Among the women with a history of HDP, those who received information and guidance during pregnancy and the postpartum period showed a modest but slightly higher likelihood of being in the action (20.0% vs. 17.4%) and maintenance (16.0% vs. 4.3%) stages than those who did not receive any support and demonstrated a lower likelihood of being in the preparation stage (62.0% vs. 73.9%) (Figure 2d).

**FIGURE 3:**
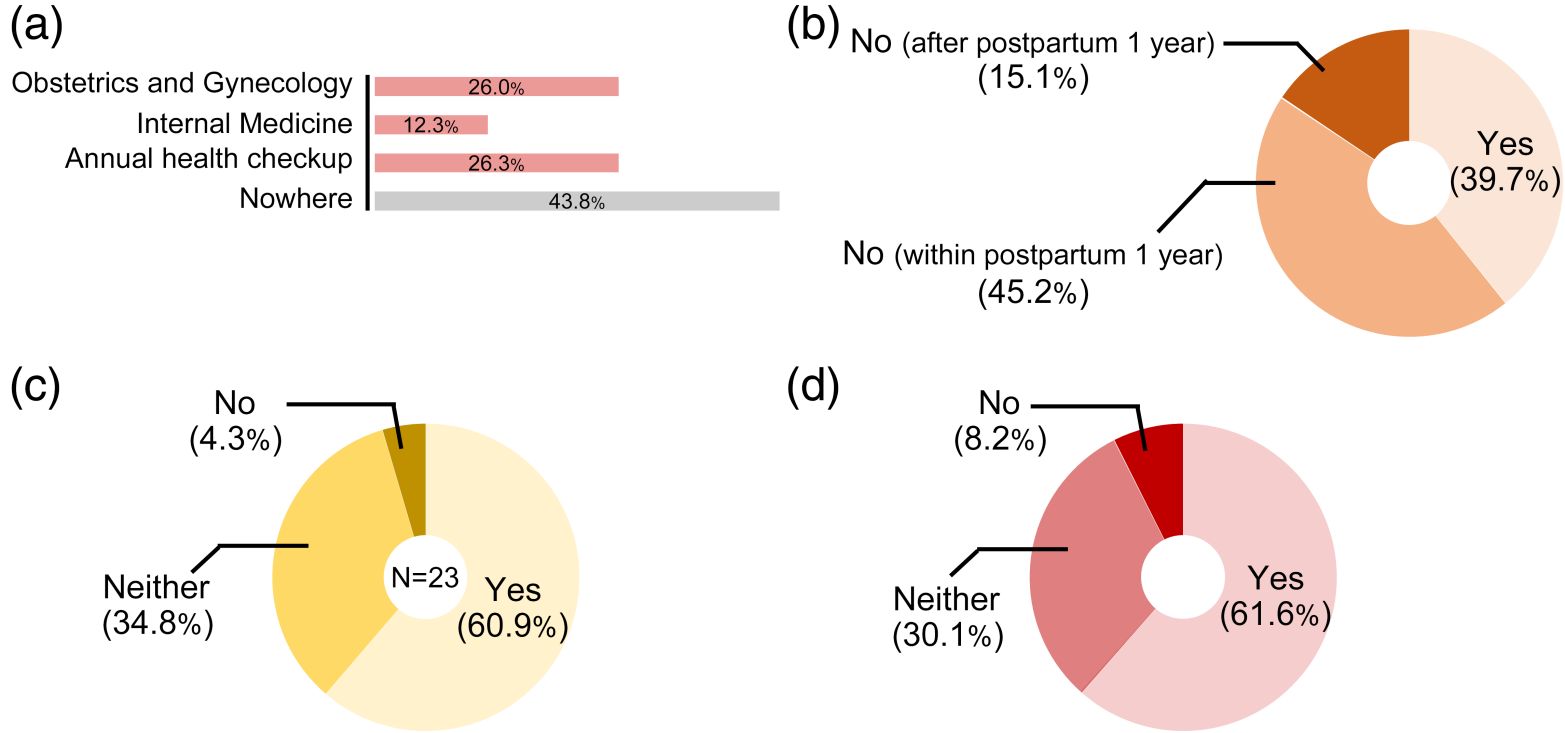
Long‐term follow‐up and interpregnancy care provided to women with a history of hypertensive disorders of pregnancy. (a) The clinic type where women with a history of hypertensive disorders of pregnancy were followed up after the 1‐month postpartum checkup. (b) The ratio of women receiving regular checkups. (c) The ratio of women who expressed wishing that they had received information and guidance during a previous pregnancy. (d) The ratio of women who expressed wanting the opportunity to discuss the next pregnancy, future risk, and lifestyle modifications with an obstetrician. Multiple answers were allowed for questions pertaining to (a).

Regarding postpartum follow‐up, 43.8% of the women with a history of HDP did not consult internal medicine or obstetrics/gynecology specialists or attend any regular checkups after their 1‐month postpartum checkups (Figure 3a). Figure 3b shows the proportion of women who attended regular checkups or specialist consultations. The proportion of women who underwent regular checkups (39.7%) was similar to that of women without a history of HDP (46.1%) (Figure 3b, Figure S1d). Among women who did not receive any information or guidance during their previous pregnancy (*n* = 23), 60.9% (*n* = 14) expressed having wanted to receive it (Figure 3c). Among women with a history of HDP, 61.6% wanted an opportunity to discuss their next pregnancy, future risks, and lifestyle modifications with an obstetrician (Figure 3d).

## DISCUSSION

The web‐based survey of this study assessed the current status of postpartum follow‐up care for Japanese women with a history of HDP. Its main findings were as follows. First, approximately one‐third of the women did not receive any information regarding their future risks or recommended preventive measures. In addition, the awareness level of HDP among these women was low, similar to that among women without a history of HDP except for that regarding HDP recurrence in the next pregnancy and the safety of antihypertensive drug use during breastfeeding. Second, approximately half of the women with a history of HDP received information and guidance from their midwives and nurses. Third, women who received information and guidance were more likely to implement behavioral changes than those who did not receive such support, indicating the importance of patient education for healthy behavioral changes. Finally, approximately 60% of the women with a history of HDP did not consult internal medicine or obstetrics/gynecology specialists or attend any regular checkups after their 1‐month postpartum checkup.

These findings highlight significant gaps in the provision of information and postpartum follow‐up care for women with a history of HDP in Japan. Despite the well‐established association between HDP and long‐term health risks, such as CVD and metabolic syndrome,^24^ our findings suggest that a substantial proportion of these women are not adequately informed and do not understand their risks and necessary preventive measures, resulting in the loss of valuable opportunities to improve their future health outcomes. This study's findings highlight the need for improved educational strategies and systematic follow‐up protocols to ensure that women are adequately informed about and supported in managing their long‐term health risks.

One of the most concerning study findings was the lack of awareness regarding long‐term health consequences among women with a history of HDP. While two‐thirds of these women were aware of the risk of HDP recurrence, approximately half were aware of the increased risk of CVD, and even fewer knew about the risks of metabolic syndrome and cognitive impairment. This lack of knowledge is concerning, as awareness is the first step toward motivation and the increased possibility of effective risk management and prevention.^20^ Without a proper understanding of their disease risk, patients cannot engage in health‐seeking behaviors that can mitigate their risks. In addition, awareness levels, except for the risk of HDP recurrence and safety of antihypertensive drug use, were comparable between women with versus without a history of HDP. Women who received information and guidance from their healthcare providers did not sufficiently understand their HDP‐related risks, indicating the need to review the method and contents of the information provision and guidance processes and the re‐education timing for patients in each facility.

The Stages of Change model, also known as the transtheoretical model, is a psychological theory that describes the process by which individuals change their behaviors.^23^ This model is widely used in various fields, including health education, addiction treatment, and psychotherapy, to help individuals understand and navigate the behavioral change process. Notably, most women with a history of HDP in this study remained in the preparation stage in both groups even after receiving information and guidance. However, a modest shift toward the action and maintenance stages was observed in the guided versus non‐guided group (i.e., combination of lower contemplation and preparation rates, slightly higher action rate, and higher maintenance rate in the guided versus non‐guided group). This finding, related to the stages of behavioral change, deserves special emphasis in the patient education process.

The low awareness rate (12.3%) of preventive measures such as the use of low‐dose aspirin further emphasizes the need for enhanced education about effective interventions to prevent HDP recurrence, particularly preeclampsia. Importantly, despite sufficient evidence of prevention through low‐dose aspirin use, a significant risk reduction was observed in a previous study among a subgroup in which aspirin was initiated at ≤16 weeks' gestation.^25^ Therefore, the lack of this information among women with a history of HDP may lead to missed opportunities to initiate low‐dose aspirin therapy in a timely manner. However, low awareness rate of low‐dose aspirin must be interpreted cautiously because our study did not distinguish between HDP subtypes. Low‐dose aspirin is recommended for the prevention of preeclampsia but not gestational hypertension in Japan. Moreover, the study reported that a substantial proportion of patients (56.2%) was unaware that most antihypertensive medications are safe during breastfeeding. This lack of knowledge can lead to the unnecessary cessation of breastfeeding, thereby depriving both mothers and infants of its benefits. Conversely, concerns about the potential adverse effects of antihypertensive medications may lead some women to discontinue their use, which could result in negative health outcomes. Therefore, healthcare providers must ensure that critical information is effectively communicated to postpartum women.

A scoping review published in 2019 on women's knowledge of the link between HDP and CVD revealed a global trend toward insufficient awareness.^26^ Six of seven international studies reported that most women were unaware of their increased risk after HDP.^26^ In the United States, a reported 71% of women are unaware of the link between preeclampsia and CVD.^27^ Similar findings were observed in Portugal, Canada, and the United Kingdom.^28^, ^29^, ^30^ These studies' conclusions align with our findings, highlighting a significant gap in patient education regarding long‐term health risks after HDP. In contrast, an Australian study showed higher awareness, with most women recognizing the future risks of hypertension (96%) and stroke (66%).^31^

One notable finding of the current study was that one‐third of the participants reported receiving no information from their healthcare providers about their long‐term health risks. This lack of information is particularly concerning given the importance of informed patient engagement in managing lifestyle diseases. Although the underlying causes of this issue are not fully understood, they may be partly attributed to challenges within the healthcare system (e.g., a high patient‐to‐physician ratio, especially in primary care facilities, and limited human resources for multidisciplinary care teams) or a lack of awareness among healthcare providers.^32^ One potential solution to the lack of awareness among healthcare providers is the “Health Care Provider System for HDP in Japan” established by the Japan Society for the Study of Hypertension in Pregnancy in 2023.^33^ Participation in HDP healthcare provider training sessions equips them with the knowledge and skills necessary to provide comprehensive support to women with a history of HDP and their families.

This study's findings have important implications for midwives and nurses who provide information and education during the postpartum period. The fact that 46.6% of women reported receiving information from midwives or nurses highlighted their potential to play a crucial role in patient education and bridge the knowledge gap, particularly in healthcare settings with high patient‐to‐physician ratios and limited physician time in primary care facilities. Although task shifting and sharing are promising strategies to address healthcare workforce shortages and simultaneously achieve a multidisciplinary care approach, the importance of an educational system for midwives and nurses should be emphasized.

More than half of the women with a history of HDP in this study did not attend regular checkups or consult specialists after their 1‐month postpartum visit. According to the Guidelines for Obstetrical Practice in Japan 2023 edition, the current status of follow‐up care reported by the participants is unsatisfactory for monitoring and managing their long‐term risks. However, among the 44 women who did not receive regular checkups after the 1‐month postpartum visit, 33 delivered within 1 year of the survey (data not shown). Therefore, we could not conclude that the follow‐up practice was insufficient in Japan. Further research is needed to evaluate the frequency of regular medical checkups received during the postpartum period.

This study's findings have two important implications for postpartum care practices and the management of women with a history of HDP. First, an improved patient education system is urgently needed to address long‐term HDP‐associated risks. Healthcare providers, including obstetricians, midwives, and nurses, must ensure that women with a history of HDP are fully informed of the risks and available preventive measures. The development and dissemination of patient educational materials (e.g., videos, applications, and pamphlets) and incorporation of dedicated counseling sessions (e.g., obstetricians and internists) into routine postpartum care may address this knowledge gap in the field of obstetrics.^34^ However, educational materials and routine systematized counseling for women with HDP are currently unavailable in Japan.

Second, uninterrupted postpartum follow‐up care protocols should be established and implemented for women with a history of HDP. Such protocols include regular health checkups with specialists or primary care physicians and targeted interventions to monitor and manage patients' blood pressure, metabolic health, and kidney function.^14^, ^15^ However, there is insufficient evidence regarding whether such a comprehensive approach can effectively reduce future CVD and metabolic disease risks.

The strengths of this study are as follows. First, to our knowledge, this is the first study to evaluate the current status of postpartum follow‐up care in Japan and patient awareness using questionnaires. Second, it evaluated the comparative awareness of HDP‐related risks and preventive measures among women with versus without a history of HDP.

This study had several limitations. First, the sample size of women with a history of HDP was small (*n* = 73). In addition, the participants were recruited through an application from pregnant and postpartum women but not from the general population, which may have introduced selection bias. Thus, further studies are required to confirm the generalizability of our findings. Second, although we evaluated postpartum care from the patient's perspective, we must still evaluate it from the healthcare provider's perspective. Third, we could not obtain detailed information about the participants' background factors affecting their awareness levels, such as facility level (primary or tertiary hospital), type of HDP (early‐ or late‐onset, preeclampsia, or gestational hypertension), anthropometric measurements (e.g., body weight and body mass index), and family history.^26^, ^30^, ^35^, ^36^ Finally, we could not explore the barriers to information provision for women with a history of HDP. Understanding such barriers will provide solutions for implementing uninterrupted postpartum follow‐up care. One possible barrier is that some healthcare providers, including obstetricians, midwives, and nurses, lack knowledge and are not adequately trained or prepared to provide the required patient education. Further research is required to assess the knowledge gaps among healthcare providers in Japan regarding future HDP‐related risks.

In conclusion, this study highlights the lack of awareness of long‐term HDP‐related risks and the insufficient information provided by healthcare professionals during the prenatal and postpartum periods. This study's findings may help improve structured patient education programs and follow‐up postpartum care protocols. Additionally, task shifting and sharing with midwives and nurses are important for addressing the current issues in Japan. The postpartum period offers a unique and valuable opportunity to educate women about their health risks and implement strategies aimed at reducing their future health complications.

## AUTHOR CONTRIBUTIONS

**Takafumi Ushida:** Conceptualization; data curation; formal analysis; funding acquisition; investigation; methodology; project administration; writing – original draft. **Sho Tano:** Investigation; writing – review and editing. **Seiko Matsuo:** Investigation; writing – review and editing. **Kazuya Fuma:** Investigation; writing – review and editing. **Kenji Imai:** Investigation; supervision; writing – review and editing. **Hiroaki Kajiyama:** Supervision; writing – review and editing. **Tomomi Kotani:** Conceptualization; supervision; writing – review and editing.

## CONFLICT OF INTEREST STATEMENT

Hiroaki Kajiyama, the editor‐in‐chief of Journal of Obstetrics and Gynecology Research and the co‐author of this article, was excluded from editorial decision‐making related to the acceptance and publication of this article. An associate editor independently handled the editorial decision‐making to minimize bias. The authors declare no conflicts of interest relevant to this research.

## Supporting information


**Figure S1.** Patient awareness of hypertensive disorders of pregnancy and the feasibility of lifestyle modifications, stages of behavioral change, and postpartum follow‐up among women without a history of hypertensive disorders of pregnancy. (A) Patient awareness of hypertensive disorders of pregnancy. (B) Items about feasible lifestyle modifications during child‐rearing in women without a history of hypertensive disorders of pregnancy. (C) Stages of behavioral change among women without a history of hypertensive disorders of pregnancy. (D) Ratio of women who attended regular checkups after the 1‐month postpartum checkup. Multiple answers were allowed for questions pertaining to B.

**Data S1.** The questionnaire administered to women with a history of hypertensive disorders of pregnancy.

**Data S2.** The questionnaire administered to women without a history of hypertensive disorders of pregnancy.

## Data Availability

All data supporting the findings of this study are disclosed in the article and Data S1 and S2.
