# Supplementary figures and images for: Patient awareness of long‐term cardiovascular and metabolic disease risks after hypertensive disorders of pregnancy in Japan
Source: J Obstet Gynaecol Res. 2024 Dec 11;51(1):e16183. doi: 10.1111/jog.16183 (PMC11634531; doi:10.1111/jog.16183)

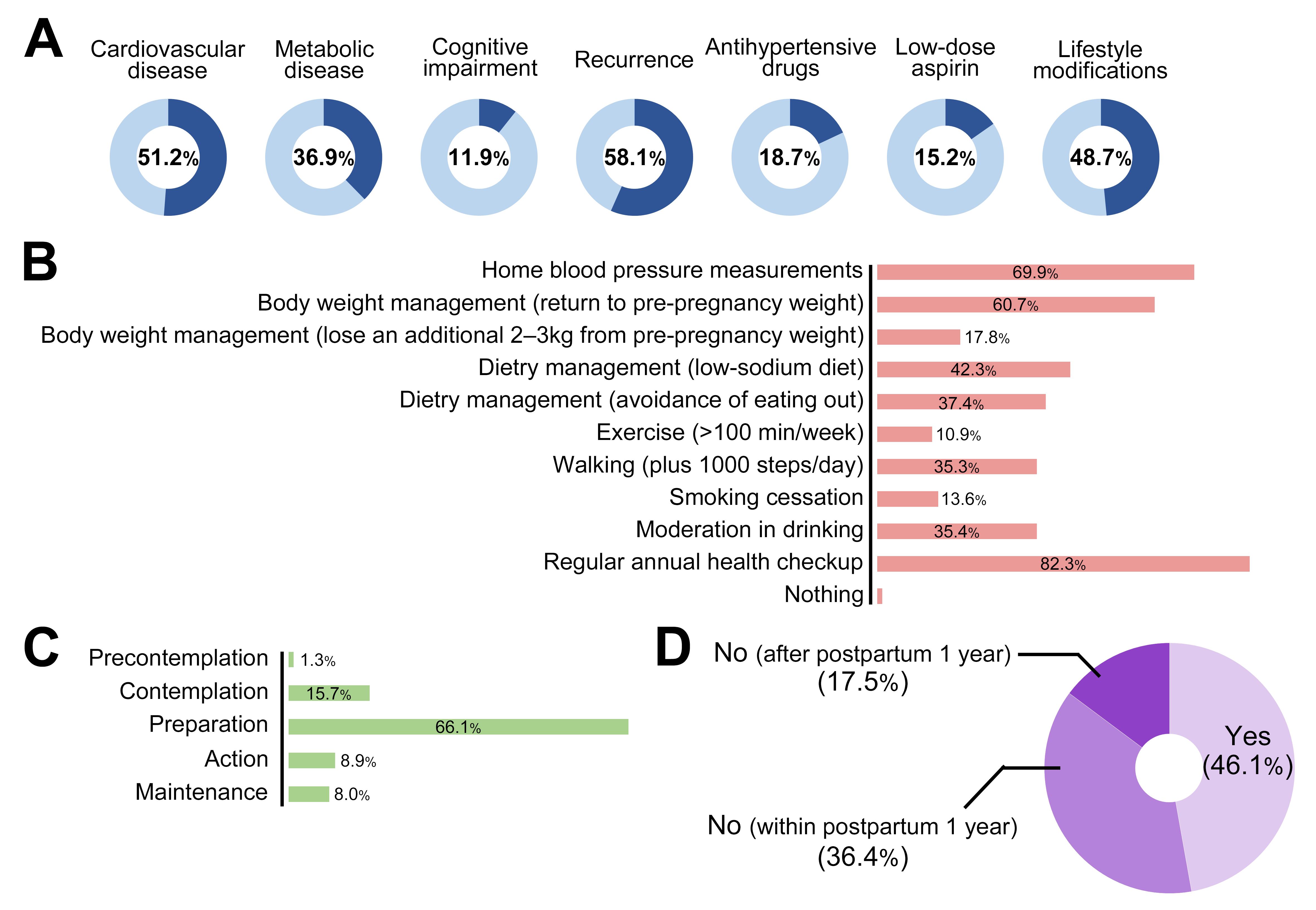

Supplement: Supplementary file 1 — Figure S1. Patient awareness of hypertensive disorders of pregnancy and the feasibility of lifestyle modifications, stages of behavioral change, and postpartum follow‐up among women without a history of hypertensive disorders of pregnancy. (A) Patient awareness of hypertensive disorders of pregnancy. (B) Items about feasible lifestyle modifications during child‐rearing in women without a history of hypertensive disorders of pregnancy. (C) Stages of behavioral change among women without a history of hypertensive disorders of pregnancy. (D) Ratio of women who attended regular checkups after the 1‐month postpartum checkup. Multiple answers were allowed for questions pertaining to B. [file JOG-51-0-s003.tif]
