# Supplementary material for: Patient awareness of long‐term cardiovascular and metabolic disease risks after hypertensive disorders of pregnancy in Japan
Source: J Obstet Gynaecol Res. 2024 Dec 11;51(1):e16183. doi: 10.1111/jog.16183 (PMC11634531; doi:10.1111/jog.16183)
Supplement: Supplementary file 2 — Data S1. The questionnaire administered to women with a history of hypertensive disorders of pregnancy. [file JOG-51-0-s001.docx]

Section 1- About you and diagnosis of hypertensive disorders of pregnancy

Q1: Do you provide consent to participate in the survey? Please answer after reading the informed consent form for this study.

Yes n=73

No n=0

Q2: Women who experienced childbirth were eligible for the survey. Have you ever given birth?

Yes n=73

No n=0

Q3: What is your age (years)?

18–19 n=0

20–29 n=8

30–39 n=50

40– n=15

Q4: Have you ever been diagnosed with hypertensive disorders of pregnancy?

*Hypertensive disorders of pregnancy refer to a condition in which high blood pressure occurs during pregnancy (systolic blood pressure ≥140 mmHg or diastolic blood pressure ≥90 mmHg).

Yes n=73

No n=0

Unsure n=0

Q5: Please provide the type of hypertensive disorders of pregnancy.

*If you have been diagnosed with hypertensive disorders of pregnancy several times, please provide information regarding your last pregnancy.

1. Gestational hypertension (high blood pressure during pregnancy, without other problems)

n=40

2. Preeclampsia (high blood pressure during pregnancy, with other problems, such as urinary protein) n=18

3. Chronic hypertension (high blood pressure before pregnancy or 20 weeks of gestation)

n=1

4. Superimposed preeclampsia (high blood pressure or urinary protein before pregnancy, with other problems, such as urinary protein or high blood pressure)

n=0

5. Unsure n=14

Section 2: Your knowledge of future risks and the information and guidance received from healthcare providers.

Q6: Women with a history of hypertensive disorders of pregnancy have an increased risk of hypertension or cardiovascular disease in later life, even if their blood pressure returns to normal after delivery.

Were you aware of this?

Yes n=41

No n=32

Q7: Women with a history of hypertensive disorders of pregnancy have an increased risk of diabetes mellitus or metabolic syndrome in later life.

Were you aware of this?

*Metabolic syndrome refers to a condition in which high blood pressure, high blood sugar levels, abnormal cholesterol levels, and excess body fat around the waist are present.

Yes n=27

No n=46

Q8: Women with a history of hypertensive disorders of pregnancy have an increased risk of cognitive impairment in later life.

Were you aware of this?

*Cognitive impairment refers to a decline in cognitive functions, such as memory, learning, and thinking, due to various factors that impair daily and social life.

Yes n=3

No n=70

Q9: Women with a history of hypertensive disorders of pregnancy have an increased risk of disease recurrence in subsequent pregnancies.

Were you aware of this?

Yes n=51

No n=22

Q10: Most antihypertensive drugs can be safely taken during breastfeeding.

Were you aware of this?

Yes n=32

No n=41

Q11: Low-dose aspirin during pregnancy is indicated to prevent recurrence in women with a history of hypertensive disorders of pregnancy, especially preeclampsia.

Were you aware of this?

Yes n=9

No n=64

Q12: Lifestyle modifications are important for women with a history of hypertensive disorders of pregnancy to reduce the risk of future cardiovascular and metabolic diseases.

Were you aware of this?

Yes n=42

No n=31

Q13: What kind of information regarding hypertensive disorders of pregnancy was provided by the healthcare providers at your maternity facility?

*Multiple choices are possible.

1. Future risk of hypertension and cardiovascular diseases

n=20

2. Future risk of metabolic syndrome n=9

3. Recurrence risk of hypertensive disorders of pregnancy

n=32

4. Most antihypertensive drugs can be safely taken during breastfeeding

n=19

5. Low-dose aspirin during pregnancy is indicated for recurrence prevention

n=6

6. What to do for the next pregnancy n=17

7. No information or guidance was provided at all from healthcare providers

n=23

Q14: What kind of guidance regarding lifestyle modifications was received from healthcare providers at your maternal facility?

*Multiple choices are possible.

1. Home blood pressure measurements n=52

2. Body weight management (return to pre-pregnancy weight or reduce weight to standard weight)

n=13

3. Dietary guidance (low-sodium diet, avoidance of eating out, and avoidance of cholesterol-rich foods) n=22

4. Exercise therapy (walking, running, stretching, etc.)

n=8

5. Smoking cessation n=1

6. Moderation in drinking n=1

7. Continued breastfeeding n=8

8. Regular annual health checkup n=11

9. No guidance was received at all from healthcare providers

n=13

Q15: Who gave you information about your future health risks and guidance regarding the recommended lifestyle modifications?

*Multiple choices are possible.

1. Obstetricians and gynecologists n=50

2. Physicians other than obstetricians and gynecologists (such as internists, cardiologists, and nephrologists) n=4

3. Midwives or nurses n=34

4. Nutritionists n=1

5. Pharmacists n=1

6. Self-researched n=10

7. No information or guidance was received from healthcare providers

n=10

Q16: For how long did healthcare providers at your maternal facility provide you with information about your future health risks and guidance about lifestyle modifications?

1. <5 min n=27

2. 5–10 min n=17

3. 10–15 min n=12

4. >15 min n=2

5. No information or guidance was received from healthcare providers

n=15

Q17: When did healthcare providers at your maternal facility provide you with information about your future health risks and guidance about lifestyle modifications?

*Multiple choices are possible.

1. At the time of diagnosis of hypertensive disorders of pregnancy

n=21

2. Between diagnosis and delivery n=18

3. Between delivery and discharge from your maternity facility

n=33

4. Between discharge from your maternity facility and postpartum 1-month checkup

n=23

5. No information or guidance was received from healthcare providers

n=13

Section 3- Feasibility and adoption of lifestyle modifications, your thoughts on health, and long-term follow-up

Q18: Are you following the guidance regarding the recommended lifestyle modifications that you received from healthcare providers?

*Lifestyle modifications: home blood pressure measurements, body weight management, dietary guidance, exercise therapy, smoking cessation, moderation in drinking, continued breastfeeding, and regular annual health checkup

1. Most of them n=12

2. About half of them n=20

3. Few of them n=21

4. None of them n=3

5. No guidance was received from healthcare providers

n=17

Q19: Please select lifestyle modifications that you are following now.

*Multiple choices are possible.

1. Home blood pressure measurements n=38

2. Weight loss (return to pre-pregnancy weight, or lose an additional 2–3kg from pre-pregnancy weight) n=33

3. Dietary guidance (low-sodium diet, avoidance of eating out, and avoidance of cholesterol-rich foods) n=46

4. Exercising for more than 100 minutes per week n=2

5. Smoking cessation (only for women who smoked before pregnancy)

n=5

6. Moderation in drinking (only for women who drank alcohol before pregnancy)

n=9

7. Regular annual health checkup n=21

8. Nothing n=5

Q20: Women with a history of hypertensive disorders of pregnancy can reduce recurrence risk in the next pregnancy and future risk of cardiovascular disease by improving their lifestyle habits. What are your current thoughts on lifestyle modifications?

1. I do not want to change my lifestyle n=1

2. I need to change my lifestyle a little, but I do not want to change my lifestyle

n=1

3. I need to change my lifestyle a little and will try to change it a little in the near future

n=48

4. I changed my lifestyle a little, but I could not keep the change for a long time

n=14

5. I changed my lifestyle and continue this change n=9

Q21: Did you consult with internal medicine or obstetrics/gynecology specialists to monitor blood pressure or proteinuria or attend any regular checkups after your 1-month postpartum checkup?

*Multiple choices are possible.

1. I visit/visited the Obstetrics and Gynecology Department several times

n=19

2. I visit/visited the Internal Medicine Department (e.g., cardiology and nephrology)

n=9

3. I attend/attended health checkup n=19

4. I haven't visited any medical facility or attended health checkup after the postpartum 1-month checkup n=36

Q22: Since your most recent delivery, have you undergone blood tests (e.g., glucose, cholesterol, renal function, and liver function) at clinics, hospitals, or regular health checkups?

Yes n=29

No (within postpartum 1 year) n=33

No (after postpartum 1 year) n=11

Q23: This question is for women who did not receive information or guidance during their previous pregnancy. Did you want to receive information and guidance from healthcare providers during your previous pregnancy?

Yes n=14

No n=1

Neither n=8

Q24: Do you want an opportunity to discuss what to do for your next pregnancy, future cardiovascular and metabolic risks, and lifestyle modifications with an obstetrician?

Yes n=45

No n=6

Neither n=22
