# Supplementary material for: Patient awareness of long‐term cardiovascular and metabolic disease risks after hypertensive disorders of pregnancy in Japan
Source: J Obstet Gynaecol Res. 2024 Dec 11;51(1):e16183. doi: 10.1111/jog.16183 (PMC11634531; doi:10.1111/jog.16183)
Supplement: Supplementary file 3 — Data S2. The questionnaire administered to women without a history of hypertensive disorders of pregnancy. [file JOG-51-0-s002.docx]

Section 1- About you and diagnosis of hypertensive disorders of pregnancy

Q1: Do you provide consent to participate in the survey? Please answer after reading the informed consent form for this study.

Yes n=807

No n=0

Q2: Women who experienced childbirth were eligible for the survey. Have you ever given birth?

Yes n=807

No n=0

Q3: What is your age (years)?

18–19 n=0

20–29 n=124

30–39 n=570

40– n=113

Q4: Have you ever been diagnosed with hypertensive disorders of pregnancy?

*Hypertensive disorders of pregnancy refer to a condition in which high blood pressure occurs during pregnancy (systolic blood pressure ≥140 mmHg or diastolic blood pressure ≥90 mmHg).

Yes n=0

No n=803

Unsure n=4

Section 2- Your knowledge of the future risks of hypertensive disorders of pregnancy

Q5: Women with a history of hypertensive disorders of pregnancy have an increased risk of hypertension or cardiovascular disease in later life, even if their blood pressure returns to normal after delivery.

Were you aware of this?

Yes n=413

No n=394

Q6: Women with a history of hypertensive disorders of pregnancy have an increased risk of diabetes mellitus or metabolic syndrome in later life.

Were you aware of this?

*Metabolic syndrome refers to a condition in which high blood pressure, high blood sugar levels, abnormal cholesterol levels, and excess body fat around the waist are present.

Yes n=298

No n=509

Q7: Women with a history of hypertensive disorders of pregnancy have an increased risk of cognitive impairment in later life.

Were you aware of this?

*Cognitive impairment refers to a decline in cognitive functions, such as memory, learning, and thinking, due to various factors that impair daily and social life.

Yes n=96

No n=711

Q8: Women with a history of hypertensive disorders during pregnancy have an increased risk of disease recurrence in subsequent pregnancies.

Were you aware of this?

Yes n=469

No n=338

Q9: Most antihypertensive drugs can be safely taken during breastfeeding.

Were you aware of this?

Yes n=151

No n=656

Q10: Low-dose aspirin during pregnancy is indicated to prevent recurrence in women with a history of hypertensive disorders of pregnancy, especially preeclampsia.

Were you aware of this?

Yes n=123

No n=684

Q11: Lifestyle modifications are important for women with a history of hypertensive disorders of pregnancy to reduce the risk of future cardiovascular and metabolic diseases.

Were you aware of this?

Yes n=393

No n=414

Section 3- Feasibility of lifestyle modifications during childrearing, your thoughts on health, and long-term follow-up

Q12: Please select lifestyle modifications that you can make while raising your child. *Multiple choices are possible.

1. Home blood pressure measurements n=564

2. Body weight loss (return to pre-pregnancy weight)

n=490

3. Body weight loss (loss of an additional 2–3 kg from pre-pregnancy weight)

n=144

4. Dietary management (low-sodium diet and avoidance of cholesterol-rich foods)

n=341

5. Dietary management (avoidance of eating out) n=302

6. Exercise (>100 min/week) n=88

7. Walking (plus 1000 steps/day) n=285

8. Smoking cessation (only for women who smoked before pregnancy).

n=110

9. Moderation in drinking (only for women who drank alcohol before pregnancy)

n=286

10. Regular annual health checkup n=664

11. Nothing n=7

Q13: Improving lifestyle habits is important for health and for reducing the risk of future cardiovascular and metabolic diseases. What are your current thoughts on lifestyle modifications?

1. I do not want to change my lifestyle n=10

2. I need to change my lifestyle a little, but I do not want to change my lifestyle

n=121

3. I need to change my lifestyle a little and will try to change it a little in the near future

n=511

4. I changed my lifestyle a little, but I could not keep the change for a long time

n=69

5. I changed my lifestyle and continue this change

n=62

Q14: Since your most recent delivery, have you undergone blood tests (e.g., glucose, cholesterol, renal function, and liver function) at clinics, hospitals, or regular health checkups?

Yes n=372

No (within postpartum 1 year) n=294

No (after postpartum 1 year) n=141
